# Identification and Characterization of Enhancer-Blocking Insulators to Reduce Retroviral Vector Genotoxicity

**DOI:** 10.1371/journal.pone.0076528

**Published:** 2013-10-03

**Authors:** Amy C. Groth, Mingdong Liu, Hao Wang, Emilie Lovelett, David W. Emery

**Affiliations:** 1 Department of Medicine, Division of Medical Genetics, University of Washington, Seattle, Washington, United States of America; 2 Department of Genome Sciences, University of Washington, Seattle, Washington, United States of America; 3 Institute for Stem Cell and Regenerative Medicine, University of Washington, Seattle, Washington, United States of America; University of Birmingham, United Kingdom

## Abstract

The chromatin insulator cHS4 can reduce silencing chromosomal position effects and genotoxicity associated with integrating viral vectors. However, the fully active version of this element can also reduce vector titers and is only partially effective. In order to identify alternatives to cHS4, we developed a functional lentiviral vector-based reporter screen for enhancer-blocking insulators. Using this system, we screened candidate sequences that were initially identified by chromatin profiling for binding by CTCF and for DNase hypersensitivity. All 12 analyzed candidates blocked enhancer-promoter activity. The enhancer-blocking activity of the top two candidates was confirmed in two complementary plasmid-based assays. Studies in a gammaretroviral reporter vector indicated these two candidates have little to no effect on vector titers, and do not diminish vector expression in primary mouse bone marrow cultures. Subsequent assessment in a mouse *in vivo* tumor formation model demonstrated that both candidates reduced the rate of gammaretroviral vector-mediated genotoxicity as effectively as the cHS4 insulator. In summary, we have developed a novel lentiviral vector-based method of screening candidate elements for insulator activity, and have used this method to identify two new insulator elements capable of improving the safety of retroviral vectors without diminishing vector titers or expression. These findings expand the limited arsenal of insulators functionally validated to reduce the rate of retroviral vector-mediated genotoxicity.

## Introduction

Chromatin insulators have been shown to improve both the expression and safety of integrating viral vectors [1]. To date, most studies of insulators in gammaretroviral and lentiviral vectors have focused on the prototypical chromatin insulator cHS4, which was derived from DNase Hypersensitive Site (DHS) 4 of the chicken β-globin locus control region [2]. This element acts as a barrier insulator capable of reducing the silencing of viral vectors by chromosomal position effects [3-5], and an enhancer-blocking insulator capable of reducing the activation of cellular proto-oncogenes by vector enhancers [6-8]. The enhancer-blocking activity of this and other chromatin insulators from higher eukaryotes is associated with the zinc-finger DNA-binding protein CCCTC-binding factor (CTCF) [9-11], and the presence of DHSs [12,13]. Enhancer-blocking insulators are thought to function through physical CTCF-mediated interactions between adjacent insulators or through CTCF-mediated tethering of chromatin fibers to structural elements within the nucleus [11,14]. Although several enhancer-blocking insulators have been described in the literature, only the cHS4 element, alone or in combination with other elements, has been shown directly to prevent genotoxicity associated with gammaretroviral and lentiviral vector transduction [7,8,15,16]. However, this activity has only been demonstrated in studies using a combination of the CTCF binding sites from the cHS4 and BEAD-1 insulators that have no barrier insulator activity [16], or the full-length 1.2 kb version of the cHS4 insulator that has been shown to reduce vector titers in several settings [1,8]. Although small binding cores for CTCF or the transcription factor nuclear factor-1 (NF1) can also block enhancer-promoter interactions [17], this activity requires the use of tandem repeats of 6-7 copies, an arrangement that is known to cause deletional recombination in gammaretroviral and lentiviral vectors [18,19]. Efforts have been made to determine the minimum sequences of the originally characterized 1.2 kb cHS4 insulator that are necessary for full insulator activity, yet no smaller fragments have been shown to reduce vector-mediated genotoxicity in rigorous *in vivo* tumor models. Indeed, a smaller 250 bp core from the cHS4 element with suboptimal enhancer-blocking activity not only failed to prevent malignant transformation in a clinical trial, but is thought to have played a role in the transformation event [19].

With the goal of expanding the arsenal of validated chromatin insulators for reducing vector-mediated genotoxicity, we used chromatin profiling data to identify 12 potential insulator elements from the human genome, and then characterized these elements for enhancer-blocking insulator properties using *in vitro* and *in vivo* assays. In order to screen these candidates for enhancer-blocking activity, we developed a rapid and quantitative functional insulator assay based on a green fluorescent protein (GFP)-expressing lentiviral reporter vector. A proof-of-principle screen with this assay identified enhancer-blocking activity associated with all 12 candidates. We report here that two of these insulators are capable of reducing vector-mediated genotoxicity *in vivo*, without adversely affecting vector expression or titer, increasing the very limited number of insulators validated for this purpose. These studies also suggest novel properties of chromatin insulators, including the apparent dependence of this activity in some cases on orientation, and the unexpected observation that CTCF binding may also convey modest levels of barrier insulator activity.

## Materials and Methods

### Ethics Statement

All studies with mice were carried out in strict accordance with the recommendations in the Guide for the Care and Use of Laboratory Animals of the National Institutes of Health. The protocols were approved by the Institutional Animals Care and Use Committee at the University of Washington (Animal Welfare Assurance No. A3464-01), including criteria used to monitor for tumor formation, and methods for animal euthanasia. All efforts were made to minimize suffering.

### Plasmid-based colony assay for enhancer-blocking insulators

Candidate insulator and control sequences were cloned into the neomycin phosphotransferase (Neo) reporter plasmid pJC5-4/P4P2K [20]. This construct is based on the Neo reporter plasmid pJC5-4 that was initially used to characterize the cHS4 chromatin insulator [3]. It contains an expression cassette for Neo that is transcribed from an ^A^γ-globin gene promoter and HS2 enhancer. Insert sequences were amplified by PCR and inserted both upstream and downstream of the transcription cassette, using the Multisite Gateway® clonase system (Invitrogen Corp., Carlsbad, CA), so as to block access to the enhancer [20]. Plasmid constructs were linearized and transfected into K562 cells using the Amaxa Biosystems Nucleofector II electroporator and Nucleofector V kit (Lonza Group Ltd, Basel, Switzerland) following the manufacturer’s directions at a dose of 2 µg plasmid per 10^6^ cells. Following electroporation, the cells were resuspended in 5 mL D8 media (Dulbecco’s modified Eagles medium (DMEM) / 8% fetal bovine serum) and cultured for 7 days without selection. The cells were then collected, counted, resuspended at a dose of 5 x10^5^ cells in 5 mL DMEM, 20% FBS, 0.8% low-melting agarose and the indicated concentrations of the neomycin drug analog G418, and plated in a 60 mm dish. These semisolid cultures were then incubated another 2-3 weeks before scoring for colony formation. K562 cells have been described previously [21], and were a gift from G. Stamatoyannopoulos.

### Lentiviral vector-based GFP reporter assay for enhancer-blocking insulators

The third-generation lentiviral vector pRRLsin.cPPT.hPgk. GFP.Wpre [22], was engineered to contain the same HS2 enhancer and ^A^γ-globin gene promoter present in the plasmid used for colony assays [3], except the enhancer was moved 5' of the promoter and the Neo reporter gene was replaced with a GFP reporter gene. Candidate insulator or control elements were amplified by PCR and inserted in the AscI restriction site between the enhancer and promoter, and the integrity and orientation of each insert was confirmed by sequencing. Vesicular stomatitis virus glycoprotien G (VSV-G)-pseudotyped vector stocks were generated by a three-plasmid transfection of 293T cells in 6-well tissue culture dishes as described [22]. Unconcentrated vector stocks were passed through 0.45 µm low protein-binding filters, and used either directly or after storing at -80 °C. Both 10 µL and 100 µL of unconcentrated vector stocks were used to transduce 10^5^ K562 cells in 2 mL D8 media with 4 µg/mL polybrene, again in 6-well tissue culture dishes. The media was changed after 24 hr culture at 37 °C, 7.5% CO_2_. After an additional 72 hr of culture the cells were collected by centrifugation and resuspended at a concentration of approximately 10^6^ cells in 2 mL D8 media containing 1.5% DMSO / 100 µM hemin in order to induce erythroid differentiation [23]. After a final 72 hr culture, the cells were collected by centrifugation, washed twice with Hank’s Buffered Saline Solution (HBSS) supplemented with 2% FBS, and analyzed for vector GFP expression on a FACScan flow cytometer (BD Biosciences, San Jose, CA) using FlowJo software. The level of expression was determined by first choosing the samples with the vector dilution that generated ≤20% GFP-positive cells (so that the majority of positive cells in the sample had only one vector copy), determining the median fluorescence for the GFP-positive cells for that sample, and then comparing it to the median fluorescence of GFP-positive cells from the no-insert vector control. The cell line 293T was acquired from American Type Culture Collection (ATCC, CRL-11268).

### Plasmid-based GFP reporter assay for enhancer-blocking insulators

Candidate insulator and control sequences were cloned into a GFP reporter plasmid. The reporter construct and cloning strategy is the same as for the drug-resistant colony assay, except that the reporter construct expresses GFP. This construct also contains a second expression cassette for the drug-resistance gene Neo transcribed from the constitutive Pgk gene promoter. Plasmid constructs were linearized and transfected into K562 cells as for the Neo colony assay, except that the cells were immediately selected in liquid culture for 7 days with 0.5 mg/mL G418 (active component) to assure all cells contained the reporter construct. The cells were then collected, washed, and analyzed by flow cytometry for reporter GFP expression. The level of expression was determined by first measuring the mean fluorescence for the GFP-positive cells in each sample, and then comparing it to the mean fluorescence of the spacer-only control vector.

### Gammaretroviral vector titer determination

Candidate insulator and control sequences were cloned into the "double-copy" position of the 3' long-terminal repeat (LTR) of the gammaretroviral reporter vector MGPN2. From this position, the insert is copied into the 5' LTR during provirus formation, effectively flanking the two internal expression cassettes [4,24]. These cassettes include a GFP gene transcribed from the viral LTR promoter, and a Neo gene transcribed from the constitutive Pgk gene promoter. Producer lines were generated by plasmid transfection of the ecotropic packaging line GP+E86 and selection of individual clones with 0.75 mg/mL G418 (active component). Vector titers were determined by collecting supernatant from subconfluent cultures of these clones, transducing naive NIH3T3 cells with titrating amounts of this supernatant, and determining the fraction of cells expressing GFP after 4-5 days of culture. Between 5 and 9 clones were analyzed for each vector, and the clones with the highest titers were used for subsequent studies.

### Mouse bone marrow-transduction assay

The gammaretroviral vectors described above were used to transduce B6xD2 F1 mouse bone marrow cells by co-cultivation on vector producer cells as described [4]. The concentrations of producer cells were chosen to target a transduction rate of approximately 30% in order to limit the vector copy number per transduced cell. Following transduction, the cells were washed with HBSS and plated at 1-2x10^4^ cells per mL in growth factor "complete" methylcellulose medium (STEMCELL Technologies Inc, Vancouver, Canada) supplemented with G418 at 0.9 mg/mL (active component). Following incubation for 7-10 days, individual colonies were picked under an inverted microscope and analyzed by flow cytometry. As previously described [5,25], the percentage of GFP-positive cells in the experimental samples was determined by subtracting the amount of background signal within the established gate of mock-transduced cells (typically set at 1%). Likewise, the GFP mean fluorescence in the experimental samples was determined by subtracting the background mean fluorescence of mock-transduced samples. As a negative control, we employed the test vector with no insert. In previous studies we determined that both a 590 bp spacer and a 790 bp spacer had no effect on the likelihood or level of vector expression in this system [5].

### 32D cell-based assay for gammaretroviral vector genotoxicity

The murine myeloid cell line 32D was cultured in D8 media containing 5% murine interleukin 3 (IL-3) culture supplement (BD Biosystems, Bedford, MA). As previously described [8], these cells were transduced with the gammaretroviral vectors described above by culturing 10^6^ cells for 24 hr in virus supernatant containing 4 µg/mL polybrene at a target multiplicity of infection of approximately 5. They were subsequently washed, split into 6 independent sub-pool cultures, and incubated as above. One subculture was used to determine the frequency of gene transfer based on GFP expression (within two days of transduction in order to minimize the influence of silencing chromosomal position effects). The remaining 5 independent cultures were expanded for 7 to 9 days, collected and washed with HBSS, counted, and injected i.v. into congenic female C3H/HeJ mice (one sub-pool per mouse, no myeloablation). The recipients were then monitored for tumor formation as previously described [8]. When tumors were detected, the mice were euthanized. Tumor-bearing mice typically presented with palpable splenomegaly. The transformation rates per transduced cells were determined as described [8]. 32D cells have been described previously [26], and were a gift from T. Papayannopoulou.

### Chromatin profiling

Genome-wide CTCF-seq and DHS mapping methods have been previously described [27,28]. The CTCF and DHS peak data used in the studies reported here are available on the UCSC Genome Browser (http://genome.ucsc.edu/). The CTCF binding was performed in 23 human cell types (GM12878, K562, HeLa-S3, HepG2, HUVEC, BJ, Caco-2, GM06990, GM12801, GM12864, GM12865, GM12872, GM12873, GM12874, GM12875, HEK293, HL-60, HMEC, HRE, NHEK, SAEC, SK-N-SH RA, and WERI-Rb-1). The DHS analysis was performed in 41 human cell types (GM12878, H1-hESC, K562, HeLa-S3, HepG2, HUVEC, MCF-7, AG04449, AG04450, AG09309, AG09319, AG10803, BJ, Caco-2, CMK, GM06990, GM12865, H7-hESC, HAEpiC, HCF, HCM, HCPEpiC, HEEpiC, HGF, HL-60, HMEC, HNPCEpiC, HRCEpiC, HRE, HRPEpiC, Jurkat, NB4, NHDF-neo, NHEK, NHLF, PANC-1, SAEC, SkMC, SK-N-SH RA, Th1, Th2). All data and details can be found on line at http://genome.ucsc.edu/.

## Results

### Drawbacks to conventional drug-resistant enhancer-blocking insulator assay

**Figure 1:**
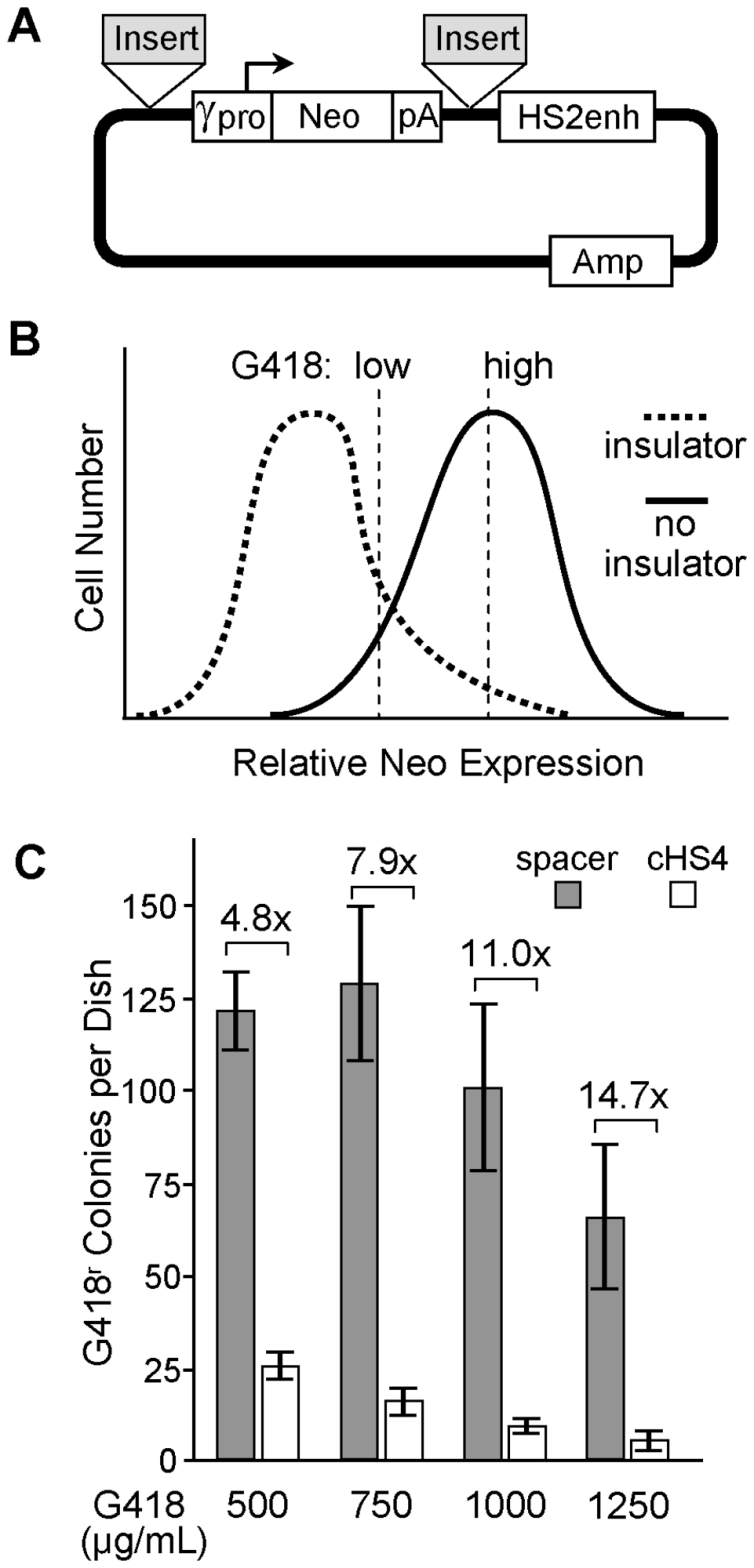
Plasmid-based colony assay is sensitive to experimental parameters. (**A**) Reporter plasmid. The reporter plasmid pJC5-4/P4-P2K contains an erythroid-specific enhancer from DHS 2 (HS2enh) of the mouse β-globin locus control region, an expression cassette for the drug-resistance gene *Neo* transcribed from the erythroid-specific Aγ-globin gene promoter (γpro), and the simian virus 40 polyadenylation signal (pA). The 1.2 kb cHS4 insulator or a 590 bp neutral spacer from the bacterial drug resistance gene *Zeo* were inserted at the two indicated locations so as to bracket the *Neo* expression cassette and separate it from the HS2 enhancer. (**B**) Schema for potential threshold effect. The theoretical range of Neo expression for the insulated (dashed line) and uninsulated spacer control (solid line) reporter constructs are shown, along with the threshold for cell survival at low and high concentrations of G418. Under this scenario, switching the concentration of G418 from low to high will have a greater impact on the rate of G418-resistant colony formation for the vector with a blocking insulator than for the vector without a blocking insulator. (**C**) Experimental data demonstrating threshold effect. The reporter plasmids were transfected into human erythroleukemia K562 cells, and then plated in semisolid low-melting agarose with the indicated concentrations of the neomycin drug analog G418. Results are shown for the colony counts per dish from 2-3 independent experiments ± standard deviation. Fold-differences between the spacer and cHS4 plasmids are indicated for each concentration of G418. *P*=0.0001 comparing data at 1000 and 1250 µg/mL G418 and *P*=0.05 comparing data at 750 and 1000 µg/mL G418 (*t*-test).

In the most widely-used functional assay for enhancer-blocking chromatin insulators, a candidate element is placed between an enhancer and a promoter linked to a Neo drug-resistance gene (Figure 1A), and the level of transgene expression is determined by measuring the rate of drug-resistant colony formation following plasmid transfection of a mammalian cell line [3]. However, because transfected plasmids form concatemers, this assay requires that the insulator candidate be placed at two locations within the reporter construct so as to fully contain the enhancer, which can complicate cloning strategies [20]. In addition, this assay does not include a ready means of controlling for variations in the underlying gene transfer rates, and the readout (colony formation) is time consuming and often difficult to score. Further, because this colony formation results in a binomial readout (reporter gene expression either does or does not exceed a certain threshold), and this threshold can be influenced by the concentration of drug used for selection (Figure 1B), the readout of this assay lacks a linear relationship to the underlying degree of enhancer-blocking. For example, as seen in Figure 1C, the difference in colony formation between constructs containing the cHS4 insulator or a neutral spacer control varied from 4.8- to 14.7-fold depending on the concentration of G418 used in the selection cultures.

### Development of a lentiviral vector-based GFP assay for enhancer-blocking insulators

**Figure 2:**
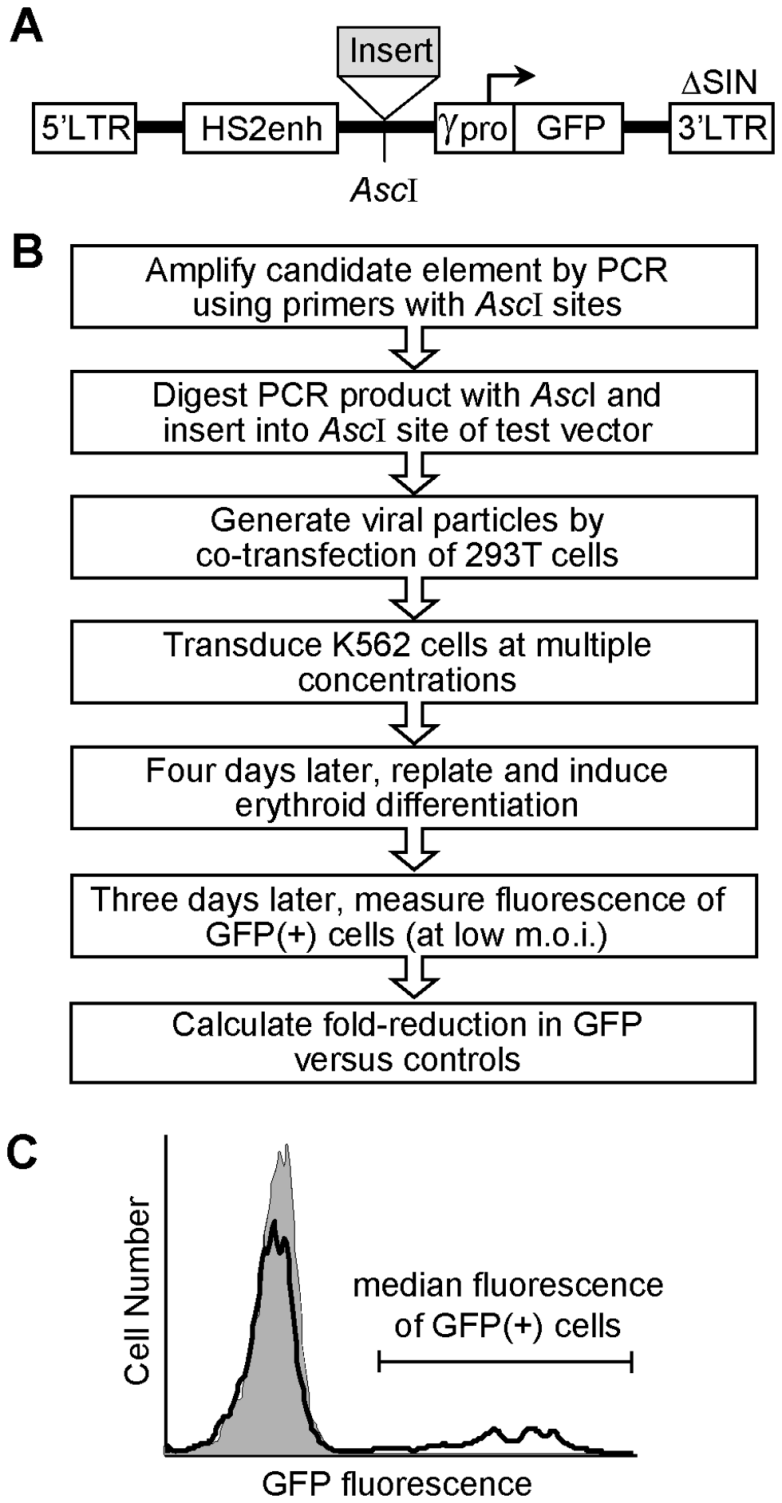
Schema and workflow for lentiviral vector-based enhancer-blocking insulator assay. (**A**) Screening vector. The lentiviral reporter vector contains the same erythroid specific HS2 enhancer and Aγ-globin gene promoter (γpro) as in the plasmid-based drug-resistant colony assay, except in this case the Neo gene is replaced with the fluorescent reporter gene GFP. The lentiviral vector is self-inactivating due to a deletion in the 3' viral long-terminal repeat (ΔSIN). An *Asc*I restriction site is situated between the enhancer and promoter for insertion of the candidate insulator elements. (**B**) Workflow for screen. See Materials and Methods for details. (**C**) Example of flow cytometric data demonstrating the general region used to determine the median fluorescence of the cells expressing vector GFP. m.o.i., multiplicity of infection.

**Figure 3:**
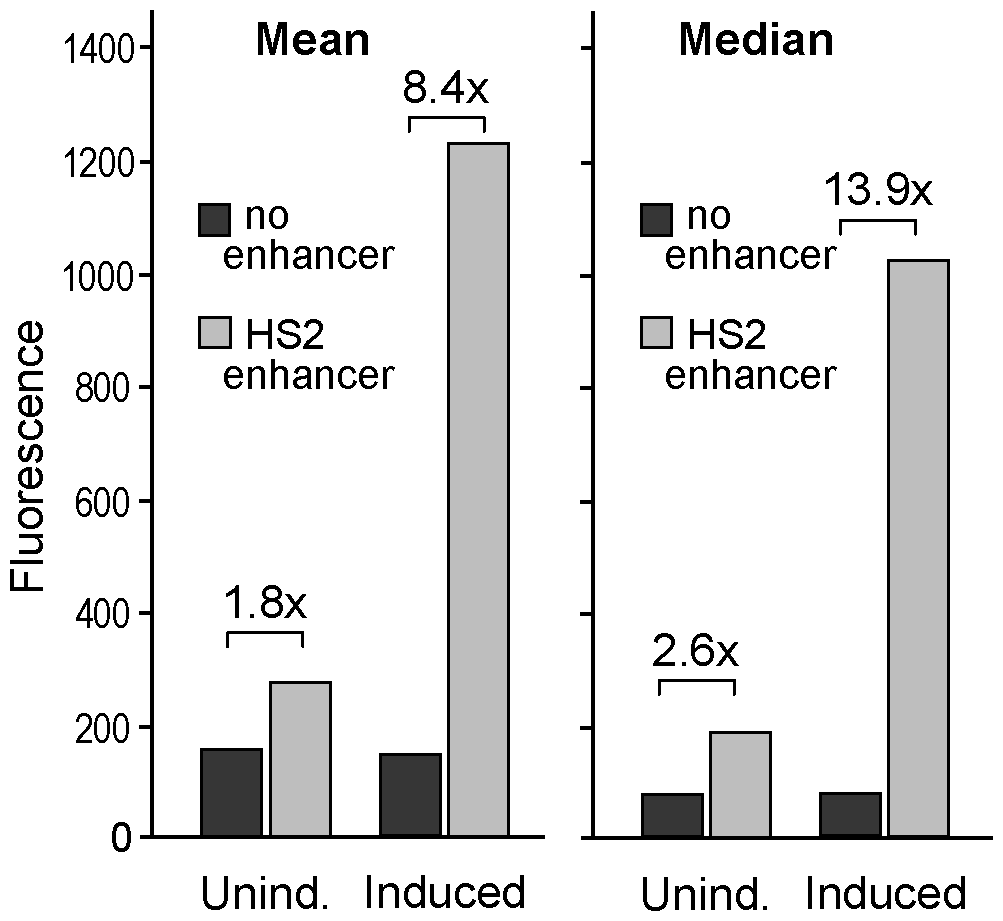
Erythropoietic induction amplifies insulator effect. K562 cells were transduced with empty (no insert) versions of the GFP-based lentiviral reporter vector containing or not containing the HS2 enhancer, cultured for the final 3 days with and without erythropoietic inducing agents as indicated, and then analyzed for GFP expression as described in the Figure 2 legend. Results are shown for both the mean and median fluorescence for the GFP-positive cells where the fraction of cells expressing GFP was ≤20%. Unind. = Uninduced. Inducing agents included 1.5% DMSO and 100 µM hemin.

As an alternative for screening candidate enhancer-blocking insulators, we developed an assay employing a GFP-based lentiviral reporter vector. As diagrammed in Figure 2A, this vector contains an expression cassette made up of the same ^A^γ-globin gene promoter and HS2 enhancer used in the plasmid-based drug-resistant colony assay, except in this case the HS2 enhancer is located 5' of the promoter, and the Neo gene is replaced with the fluorescent reporter gene GFP. As outlined in Figure 2B, candidate insulator elements are inserted between the enhancer and promoter using a rare restriction site (*Asc*I). The use of this single insertion site eliminates the need for complicated cloning strategies associate with the plasmid-based drug resistance colony assay. Virus is produced by transfection of 293T cells and used to transduce erythroid K562 cells, which are then analyzed for GFP expression. By using two different concentrations of virus supernatant at the transduction step, it is possible to routinely generate a population of cells where ≤20% of the cells are GFP-positive, indicating that they typically contain one vector copy per GFP-positive cell. By using a fluorescent reporter system, the effect of the candidate insulator on the promoter-enhancer interaction can be directly quantified using flow cytometry to measure the amount of GFP fluorescence in all of the cells that express GFP (Figure 2C). The high rate of gene transfer afforded by the lentiviral vector platform, and the resulting ability to assess vector GFP expression in a population of cells that typically contain one vector copy without drug selection, provides a ready means of accounting for the underlying rate of gene transfer. Further, we found that the dynamic range of this assay (the difference between control vectors with and without the HS2 enhancer) could be increased >4-fold by inducing the transduced K562 cells to differentiate down the erythroid lineage for three days prior to final analysis (Figure 3). It is important to note that erythropoietic induction cannot be used in the plasmid-based colony assay since such induction results in terminal differentiation and eventual apoptosis. Although these results are not unexpected given the documented activation of this enhancer during erythroid differentiation, they do serve to confirm this activity in the context of the lentiviral reporter vector. Further, these results demonstrate that the greatest dynamic range was provided by using the median level, rather than the mean level, of vector GFP expression.

**Figure 4:**
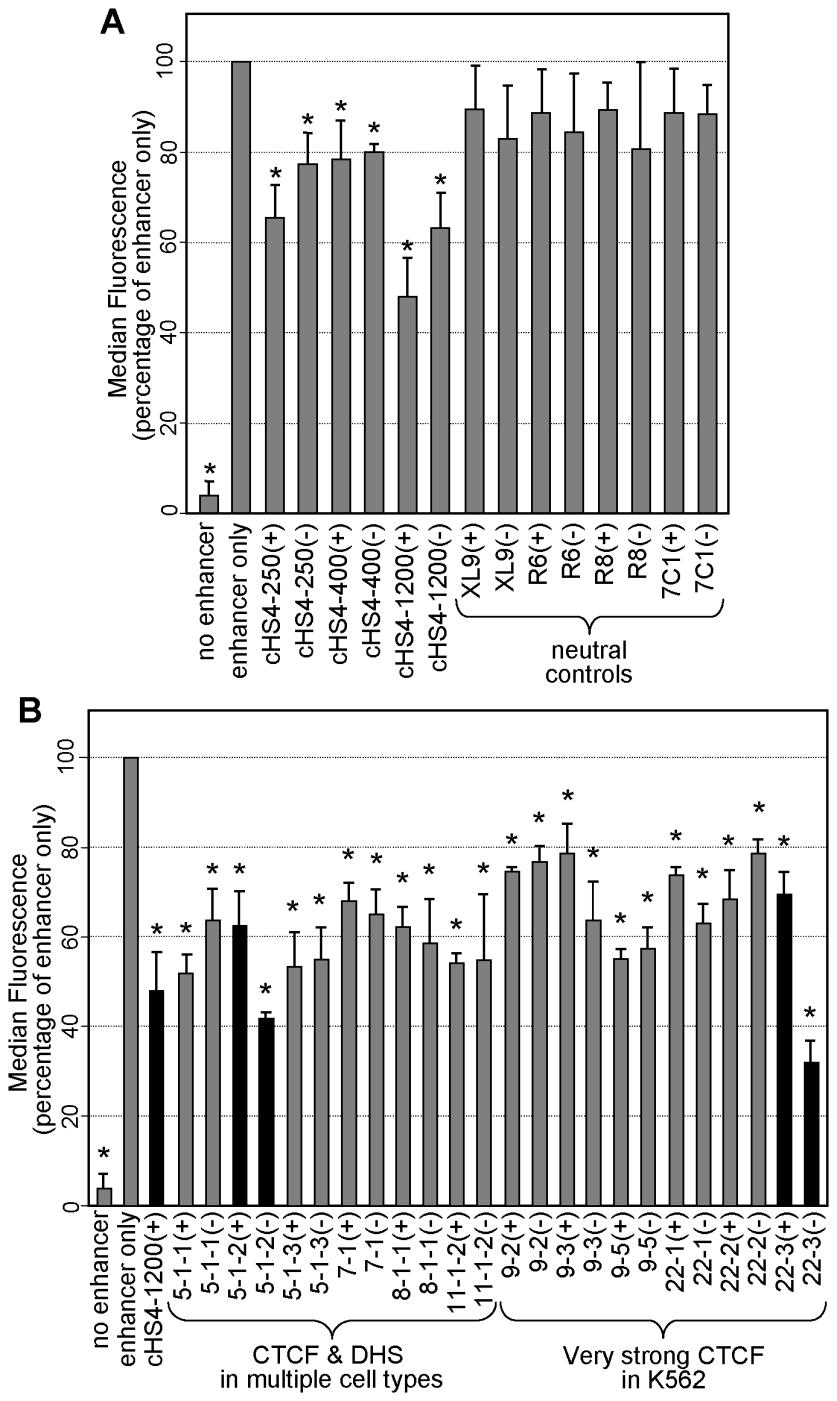
Lentiviral vector screen of candidate insulators. (**A**) Specificity of lentivector-based GFP assay. The indicated versions of the prototypical cHS4 chromatin insulator and 4 neutral control elements were inserted into the GFP-based lentiviral reporter vector and analyzed for the intensity of GFP expression in erythroid K562 cells. (**B**) Screen of candidate insulator elements. Candidate elements identified by chromatin profiling were inserted into the GFP-based lentiviral reporter vector and analyzed for the intensity of GFP expression in erythroid K562 cells as described in Figure 2. These candidates included sequences that were positive for CTCF and DHS in multiple cell types, and sequences that generated very strong peaks of CTCF binding in the erythroid cell line K562 (see Table 1 for details). Results are reported as the median fluorescence of the GFP-positive cells as a percentage of the no-insert enhancer-only control. Data represent the average ± standard deviation from 3 or more independent experiments. * *P*<0.02 versus enhancer-only (no insert) control based on *t*-test with Bonferroni correction. See Table 1 for details of control and candidate insulator fragments. (+), fragments inserted in the positive orientation based on the UCSC genome browser output; (-), fragments inserted in the negative (reverse) orientation.

In order to validate the specificity of this assay, we generated a series of lentiviral reporter vectors containing either the full-length 1.2 kb version of cHS4 used in the initial characterization of this prototypical insulator [3], a 250 bp core from the 5' end of this element that reportedly contains much of the enhancer-blocking insulator activity of the full-length fragment [12], and a 400 bp extended version of this core shown previously to contain all of the barrier insulator activity of the full-length fragment [5]. In addition, we included 4 neutral spacer controls from the human genome that are free of DHSs, conserved sequences, or binding by CTCF in K562 cells. Two of these elements were derived from "gene deserts", while the other two were derived from introns of known genes. These fragments range in size from 593-1040 bp (Table 1). As seen in Figure 4A, we found that the 1.2 kb cHS4 insulator reduced reporter GFP expression to 48 ±9% of the no-insert control in the (+) orientation, and to 63 ±8% of the no-insert control in the (-) orientation. Both the 250 bp and the 400 bp cHS4 fragments also reduced reporter GFP expression by a statistically significant amount (*P*<0.02 after Bonferroni correction), but not as well as the 1.2 kb cHS4 element. This finding is consistent with the original characterization of a single copy of the 250 bp cHS4 core [12], and provides evidence that the extended 400 bp cHS4 core may not exhibit the full enhancer-blocking activity of the 1.2 kb cHS4 element. Although all four of the neutral spacer control sequences marginally reduced vector GFP expression to an average of 87 ±14% of the no-insert control, none of these effects reached the level of statistical significance, and they presumably reflect the increased physical distance between the HS2 enhancer and ^A^γ-globin gene promoter.

**Table 1:** Candidate chromatin insulators.

**Name**	**Coordinates (hg18)**	**CTCF score^a^**	**Cell Types w/ CTCF^b^**	**Cell Types w/ DHS^c^**
**Neutral Controls (no CTCF or DHS in K562)**				
XL9	chr6:3780962-3781708	0	2 of 23	13 of 41
R6	chr6:124876143-124877183	0	1 of 23	1 of 41
R8	chr8:84272369-84273367	0	0 of 23	0 of 41
7C1	chr7:117168537-117169130	0	2 of 23	2 of 41
**CTCF & DHS in Multiple Cell Types**				
5-1-1	chr5:131427072-131428071	251	23 of 23	39 of 41
5-1-2	chr5:131427572-131428570	285	23 of 23	39 of 41
5-1-3	chr5:131428072-131429070	285	23 of 23	40 of 41
7-1-1	chr7:117142636-117143715	33	23 of 23	40 of 41
8-1-1	chr8:118943357-118944471	28	22 of 23	34 of 41
11-1-2	chr11:116166972-116168757	65	23 of 23	39 of 41
**Very Strong CTCF in K562**				
9-2	chr9:132923166-132923380	1759	23 of 23	38 of 41
9-3	chr9:133043555-133043804	1335	23 of 23	40 of 41
9-5	chr9:132911956-132912229	1676	23 of 23	41 of 41
22-1	chr22:21850661-21850897	1837	23 of 23	40 of 41
22-2	chr22:21231569-21231866	2076	23 of 23	28 of 41
22-3	chr22:18524563-18524800	1051	23 of 23	39 of 41

(a) CTCF "score" represents the highest ChIP-seq tag density within the designated region in K562 cells.

(b) Number of cell types containing one or more CTCF hot spots in the designated region from a panel of 23 cell types (see Materials and Methods for list).

(c) Number of cell types containing one or more DHS hot spots in the designated region from a panel of 41 cell types (see Materials and Methods for list).

### Screen of putative insulators with GFP-based lentiviral reporter vector

As a proof-of-principle, we used the GFP-based lentiviral vector to assess the enhancer-blocking activity of 12 candidate insulator elements identified by chromatin profiling of the human genome. We started with 6 segments that were positive for CTCF binding in 23 of 23 cell types, including K562 cells, and also contained active DHSs in 34 or more of the 41 cell types for which data is currently available, including K562 (see Table 1 for details). Three of these candidates, 5-1-1, 5-1-2, and 5-1-3, come from a region previously reported to exhibit enhancer-blocking activity by others [29]. Because this site is over 3 kb in length, we generated smaller fragments tiling across portions of the region. To identify additional candidates, we also made use of CTCF binding data in the erythroid cell line K562 [27], in part due to our interest in developing chromatin insulators for erythroid-based hemoglobinopathies [30]. We chose a total of 6 sequences with CTCF ChIP-seq tag densities in the top 0.3 percentile (Table 1). Subsequent analysis revealed that all 6 of these CTCF sites were also bound by CTCF in 23 of 23 cell types, and also contained active DHS in 28 or more of 41 cell types for which data is currently available (see Table 1 for details). The candidate elements averaged 478 bp (range 215-1115 bp). These sizes were chosen to assure that the test fragments not only contained the CTCF and DHS sites, but also included any flanking sequences and/or immediately proximal regulatory elements that could play a role in modulating their enhancer-blocking activity.

All 12 candidates were screened in both orientations using the GFP-based lentiviral reporter vector. As seen in Figure 4B, all 12 candidate elements exhibited statistically significant enhancer-blocking activity. The median level of vector GFP fluorescence for the GFP-positive cells, compared to the no-insert (enhancer-only) control, ranged from a modest 78 ±7% for candidate 9-3 in the (+) orientation to 32 ±5% for candidate 22-3 in the (-) orientation. Interestingly, there was a statistically significant orientation bias for 4 out of the 12 pairs (candidates 5-1-1, 5-1-2, 22-1, and 22-3, *P* < 0.05 by *t*-test), a property shared by the 250 bp and 1.2kb cHS4 elements (*P* < 0.02 by t-test, Figure 4A).

### Validation of the top two candidates in plasmid-based enhancer-blocking assays

**Figure 5:**
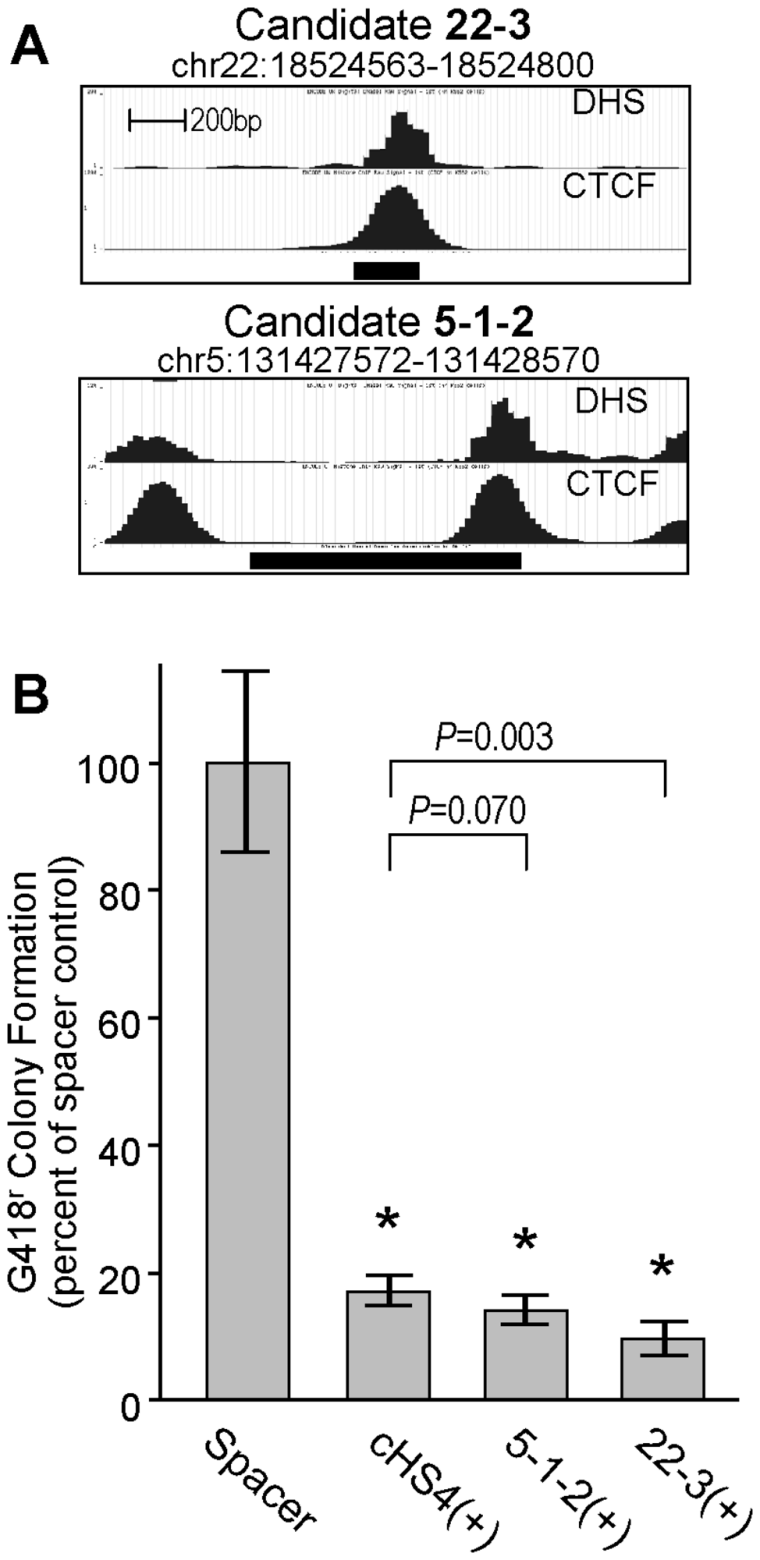
Validation of top candidate insulators in conventional drug-resistant colony assay. (**A**) Chromatin profiling for top two insulator candidates. The raw sequence tags are shown for the DHS and CTCF-binding activities over the 2 kb regions centered around the top insulator candidates 22-3 and 5-1-2 (dark horizontal bars indicate locations of candidate sequences). (**B**) Insulator activity. The top two candidate insulator elements 5-1-2 and 22-3, the 1.2 kb cHS4 insulator, or a neutral spacer (308 bp fragment from the bacterial drug resistance gene Zeo), were inserted into the reporter plasmid pJC5-4/P4-P2K (Figure 1A) in the (+) orientation and analyzed for colony formation under G418 selection (1000 µg/mL). Histograms represent the average ± standard deviation from a total of 4 samples in 2 independent experiments, and are reported as a percentage of the average colony formation obtained with the Zeo control (set at 100%). *P* values presented for comparisons between candidate elements versus the cHS4 positive control are based on *t*-test. * indicate *P*<0.001 for the cHS4 and all candidate elements versus the spacer control (*t*-test).

Although none of these candidates appeared to provide significantly more enhancer-blocking activity than the 1.2 kb cHS4 element, two elements showed nearly equivalent levels of activity: 5-1-2 and 22-3. The 999 bp candidate 5-1-2 was derived from a cluster of three CTCF-binding sites between the *IL3* and *CSF2* genes. Although this region has been shown previously to exhibit enhancer-blocking insulator activity [29], the subfragment used here does not include the 2.9 kb sequence to which the authors attributed the bulk of the activity. Candidate fragment 5-1-2 shares a dominant CTCF peak with fragment 5-1-3, but each of these fragments also contain 500 bp of unique sequences. The 237 bp candidate 22-3 is located between the genes *ZDHHC8* and *RTN4R*. The CTCF and DHS profiles for both of these insulator candidates in K562 cells is shown in Figure 5A. It is important to note that, at the resolution shown, it is not possible to determine if each CTCF peak is the result of one or more independent CTCF binding sites.

To confirm the enhancer-blocking activity of these elements, we used the conventional plasmid-based drug-resistant colony assay [3,13,20]. As diagrammed in Figure 5B, both of these candidates significantly reduced the frequency of G418-resistant colony formation compared to a neutral spacer, ranging from 6.0 ±0.8 fold for the 1.2 kb cHS4 insulator (*P*=2.5x10^-6^) to 10.8 ± 3.0 fold for the insulator candidate 22-3 (*P*=1.6x10^-6^). Comparisons between these various elements indicated that candidate 22-3 was statistically even stronger than the cHS4 insulator (*P*=0.003), although the magnitude of this difference was marginal.

**Figure 6:**
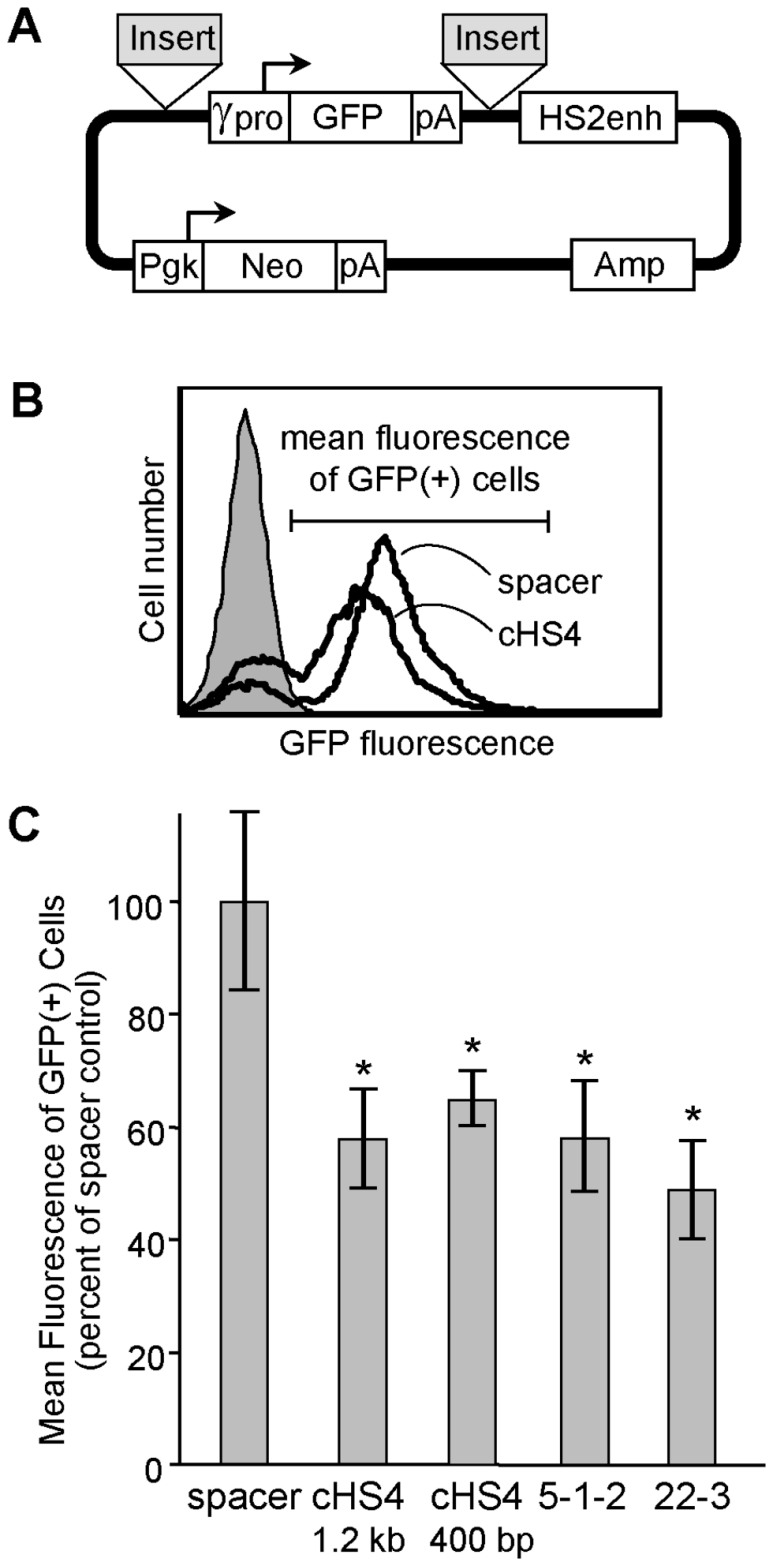
Validation of top candidate insulators in a GFP-based plasmid enhancer-blocking assay. (**A**) Reporter plasmid. The reporter construct pJC5-4/P4P2K used for the drug-resistant colony assay was modified by replacing the Neo reporter gene with a GFP fluorescent reporter gene as diagrammed. This construct also contains a second expression cassette for Neo transcribed from the constitutive Pgk gene promoter. Insulator candidate and control fragments were inserted upstream and downstream of the GFP expression cassette. (**B**) Experimental schema. Plasmid constructs were linearized and transfected into K562 cells as for the drug-resistance colony assay, and selected with low level G418 (0.5 mg/mL) in liquid cultures for 7 days. The level of reporter GFP expression was subsequently analyzed by flow cytometry using the indicated gating. (**C**) Determination of insulator activity. Histograms represent the average ± standard deviation from a total of 4 independent experiments, and are reported as a percentage of the mean fluorescence for the 308 bp Zeo spacer control (set at 100%). * *P*<0.05 versus spacer control (*t*-test).

The two-fold reduction afforded by the cHS4 insulator in the lentivector-based GFP assay (Figure 4) was far less than the 6-fold observed in the plasmid-based drug-resistant colony assay (Figure 5B) [3,5]. In order to determine whether this discrepancy was due to differences in the reporter methods or the methods of gene delivery, we analyzed two versions of the cHS4 insulator, along with the top two insulator candidates, using a GFP version of the plasmid-based insulator assay. As seen in Figure 6, we found that both the cHS4 insulator and the two insulator candidates reduced reporter GFP expression about 2-fold, from 52 ±9% of the neutral spacer control for the cHS4 insulator to 45 ±6% for candidate 22-3 (*P*<0.002). These results support our hypothesis that a fluorescence-based reporter system provides a more linear quantification of enhancer-blocking activity than the threshold-based drug-resistance assay, and provide additional evidence that the top two candidates have potent enhancer-blocking activity.

### Assessment of candidate insulator activity on gammaretroviral vector titer

**Figure 7:**
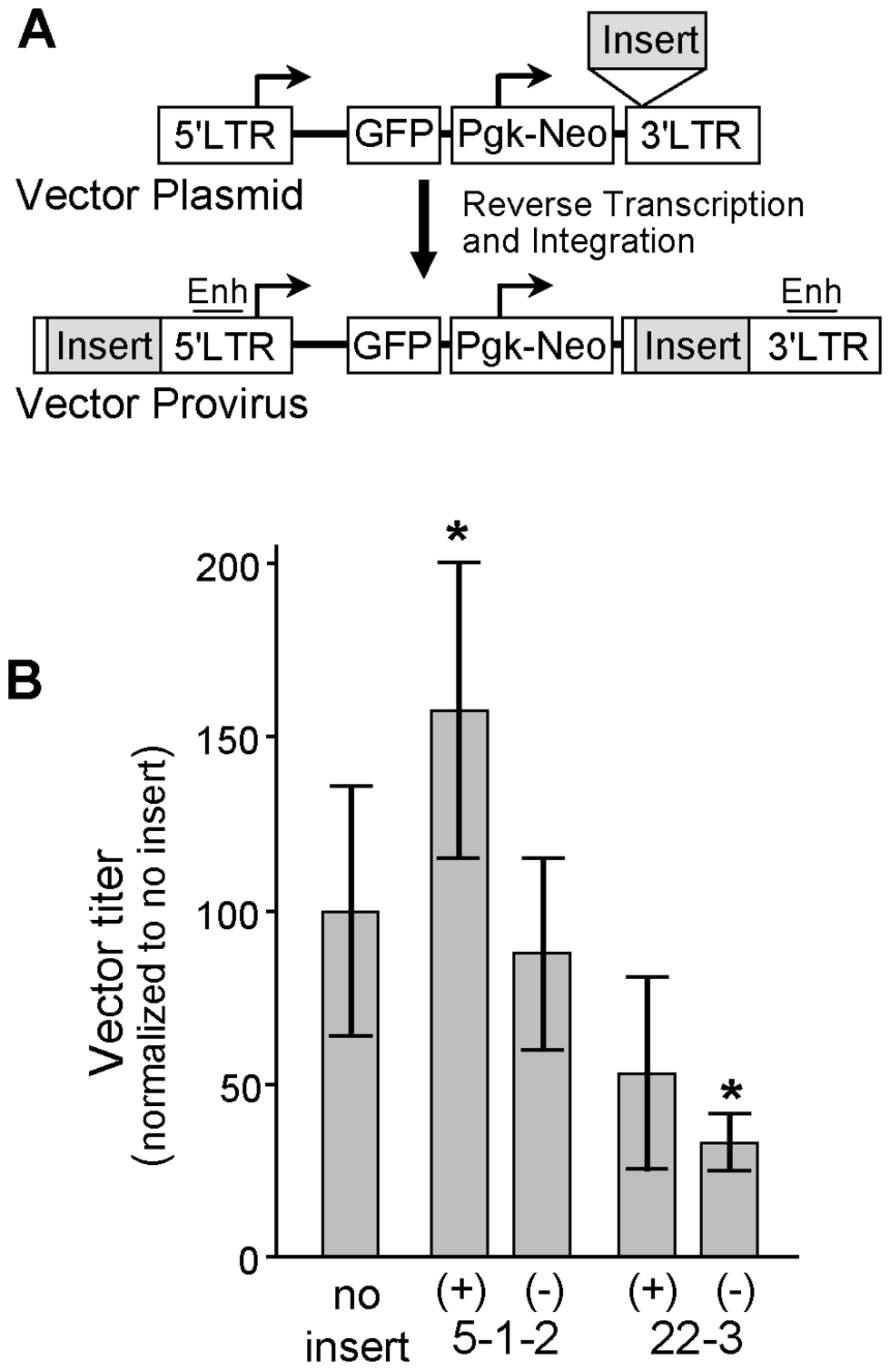
Effects of candidate insulators on gammaretroviral vector titers. (**A**) Assay vector. The gammaretroviral vector MGPN2 expresses GFP from the 5' LTR promoter, and Neo from an internal Pgk promoter. Candidate insulator and control elements are inserted into the "double-copy" position of the 3' LTR, from which they are copied into the 5' LTR during reverse transcription and provirus integration, resulting in the flanking arrangement diagrammed. (**B**) Titer data. The top two insulator candidates 5-1-2 and 22-3 were inserted into the assay vector in both the (+) and (-) orientations, and used to generate independent producer clones in the ecotropic packaging cell line GP+E86. The titer of each clone was then determined by transfer of GFP to naive NIH3T3 cells. Data is presented for five to nine independent determinations per vector, normalized to the no-insert control. Histograms indicate the average ± standard deviation. * *P*<0.05 versus no insert control (*t*-test).

The top two insulator candidates 5-1-2 and 22-3 were introduced into the "double-copy" position of the gammaretroviral reporter vector MGPN2 [4,31]. As diagrammed in Figure 7A, this results in an arrangement whereby the GFP and Neo reporter cassettes are flanked by the candidate insulators during proviral integration. Titer determinations from multiple clones demonstrated that candidate 22-3 reduced vector titers an average 2.4-fold (*P*=0.036) in the negative orientation but had no effect on vector titers in the positive orientation, and that candidate 5-1-2 increased vector titers an average 1.6-fold (*P*=0.044) in the positive orientation and had no effect on vector titers in the negative orientation (Figure 7B). For further studies we chose individual ecotropic producer clones with the titers listed in Table 2.

**Table 2:** Effects of top two candidates on viral vector activity.

**Insert**	**Highest Titer^a^**	**Expression^b^ (fold-improvement)**			**Genotoxicity^c^ (fold-reduction)**
		% GFP(+)	m.f.u.	C.V.	
None	4 x10^6^	(1.0)	(1.0)	(1.0)	(0)
cHS4(+)	2 x10^6^	2.3*	4.0*	0.58*	5.5
5-1-2(+)	4 x10^6^	1.0	1.0	0.67*	2.6
5-1-2(-)	3 x10^6^	1.7*	1.7	0.64*	3.8
22-3(+)	2 x10^6^	1.9*	3.4*	0.65*	7.9
22-3(-)	6 x10^5^	1.1	0.9	0.70*	10.2

**Figure 8:**
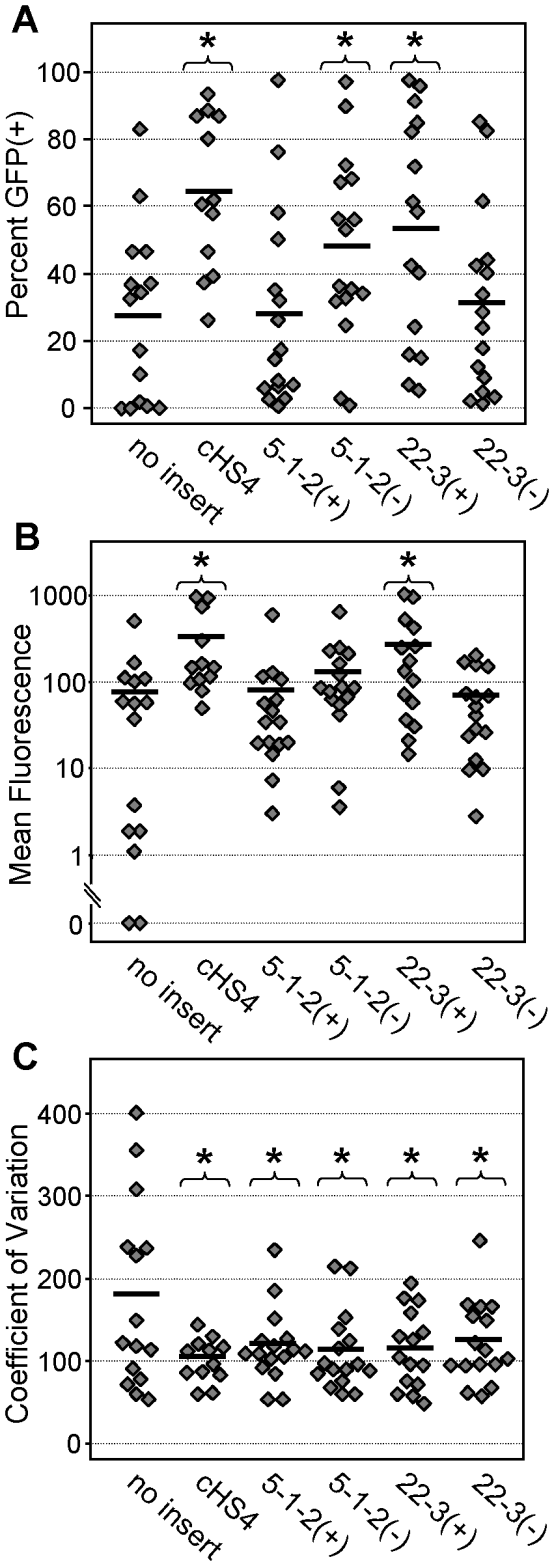
Effects of candidate insulators on gammaretroviral vector expression. Individual mouse bone marrow progenitor colonies transduced with the indicated vectors and grown under G418 selection were analyzed by flow cytometry (see Figure 7 for vector details). (**A**) Frequency of vector GFP expression reported as the percentage of cells expressing GFP. (**B**) Level of vector GFP expression reported as mean fluorescence units for cells in the GFP(+) gate. (**C**) Variation of vector expression reported as the coefficient of variation (CV) for all (ungated) cells. Each diamond represents the results from an individual clone. Data are from two independent transduced cultures. * *P*≤0.02 based on *t*-test versus the uninsulated (no-insert) control vector. Thick horizontal bars indicate the mean for all clones transduced with the indicated vector.

**Figure 9:**
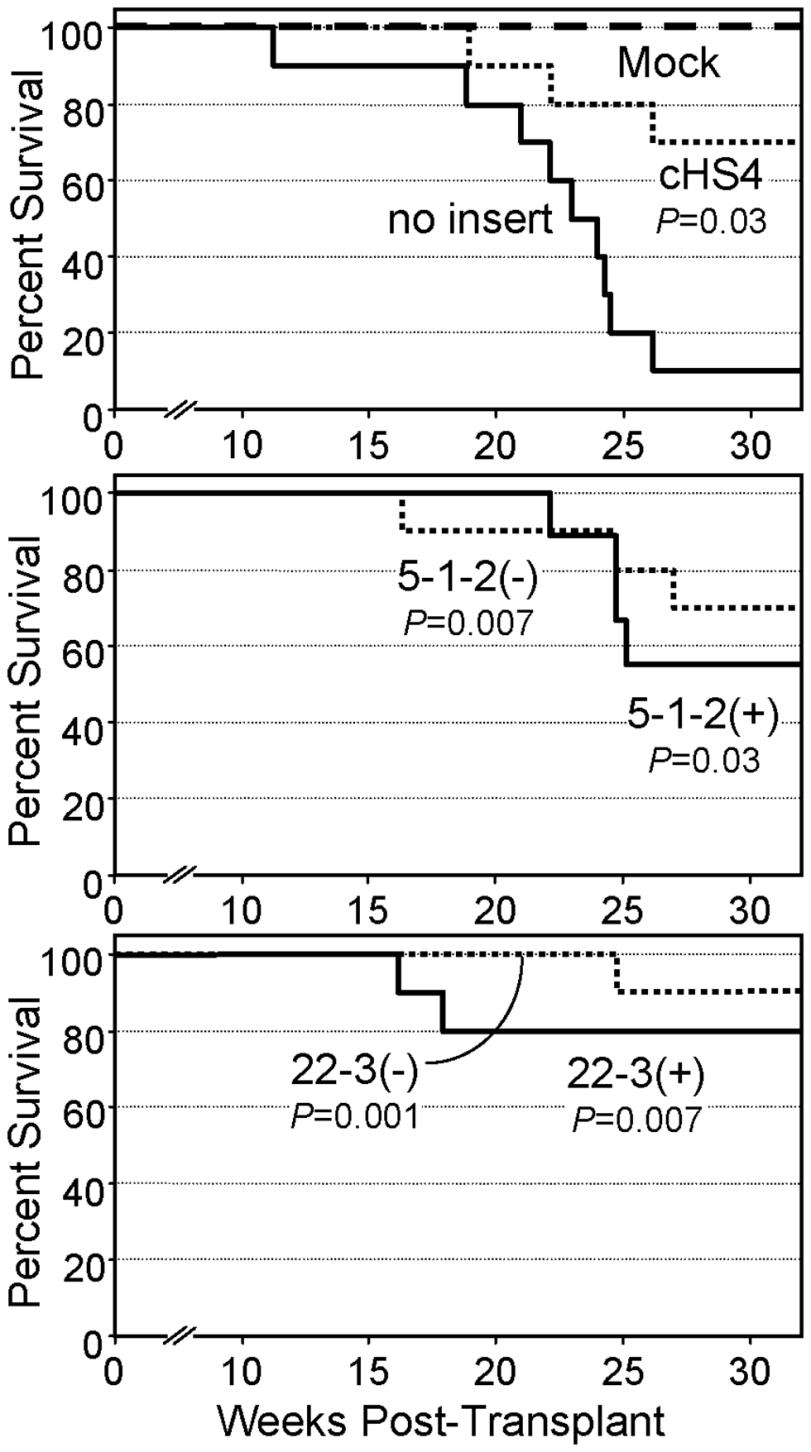
Effects of candidate insulators on gammaretroviral vector genotoxicity. Kaplan-Meier tumor-free survival curves for mice transplanted with sub-pools of vector-transduced 32D cells. 32D cells were transduced with vectors containing either no insert, the 1.2 kb cHS4 insulator, or the top two candidate insulators. Independent sub-pools were subsequently expanded and transplanted into congenic mice, which were followed for tumor formation. Mock: untransduced 32D cells. Data are from two independent experiments of 5 mice per condition each (for a total of 10 mice for each condition except for vector 5-1-2 (+), which only included 9 mice). *P* based on KS test versus the uninsulated vector.

(a) See Figure 7 for complete dataset on titers

(b) See Figure 8 for complete dataset on expression

(c) See Figure 9 for survival curves

* P < 0.05

### Assessment of candidate insulator activity on gammaretroviral vector expression

Although the initial lentiviral vector-based screen and subsequent plasmid-based assays were designed to detect enhancer-blocking insulators, these assays do not distinguish between elements that block enhancer-promoter interactions and elements that silence gene expression. Further, the informatics approach used to identify potential enhancer-blocking insulators does not preclude the co-segregation of barrier insulator activity, as seen with the cHS4 insulator. In order to address these concerns for the two top insulator candidates, we assessed the expression of the gammaretroviral reporter vectors containing these elements in primary mouse bone marrow progenitor cultures. Mouse bone marrow cells were transduced with the same vectors used to assess the effects of these candidates on vector titers at a limiting multiplicity of infection (in order to ensure a low copy number), and plated for progenitor colony formation under G418 selection in methylcellulose cultures. Individual colonies were then picked and analyzed for vector GFP expression by flow cytometry. As seen in Figure 8, and summarized in Table 2, the cHS4 element increased the fraction of cells expressing vector GFP 2.3-fold (*P*=0.0004), increased the level of vector GFP expression 4-fold (*P*=0.01), and decreased the coefficient of variation (CV) of vector GFP expression to 0.54-fold (P=0.02). These results are similar to our previous studies in this same system, and reflect the barrier insulator activity, and lack of silencing activity, of the cHS4 element [4,5,25,32]. Both candidates inserted in either orientation also served to reduce the CV to statistically lower levels, while only candidates 5-1-2 in the (-) orientation and 22-3 in the (+) orientation were able to improve the fraction of cells expressing GFP. Only candidate 22-3 in the (+) orientation was able to increase the level of vector GFP expression. These results demonstrate that the top two candidate insulators do not function as transcriptional silencers, and indeed may exhibit modest enhancer and/or barrier insulator activity.

### Assessment of candidate insulator activity on gammaretroviral vector genotoxicity

Finally, we sought to determine whether the two top candidate insulators could reduce the functional rate of retroviral vector-mediated genotoxicity. For this purpose, we utilized an *in vivo* tumor model based on the IL-3 dependent cell line 32D [8]. In this assay, 32D cells were transduced with the same gammaretroviral reporter vectors used for the titer and expression studies. Independent sub-pools of transduced cells were then expanded and transplanted into congenic mice, which were subsequently monitored for tumor formation. As diagrammed in Figure 9, both candidate elements reduced the rate of vector-mediated tumor formation compared to the uninsulated control arm (*P*<0.03). Independent studies with a vector containing a neutral spacer demonstrated that this effect was candidate-specific (manuscript in preparation). Taking into account the frequency of vector transduction, which ranged from 18-38% across the different vectors, we were able to compare the underlying transformation rates for the different candidates. As summarized in Table 2, the cHS4 control insulator reduced the rate of tumor formation 5.5-fold, consistent with our previous studies [8]. The candidate elements ranged from 2.6-fold for candidate 5-1-2 in the (+) orientation, to 10.2-fold for candidate 22-3 in the (-) orientation. Although all of the candidate elements reduced the rate of vector-mediated genotoxicity to levels well below the rate observed for the uninsulated vector (*P*=0.03 to *P*=0.001), their rates were all statistically indistinguishable from the rate observed for the vector flanked with the cHS4 insulator (P ≥ 0.67). From these studies we conclude that both of the top insulator candidates appear to be as effective as the 1.2 kb cHS4 insulator at reducing vector-mediated genotoxicity.

## Discussion

We have identified two new chromatin insulators, 5-1-2 and 22-3, which compare favorably to the prototypical insulator cHS4 for the prevention of gammaretroviral vector-mediated genotoxicity. At 999 bp, candidate 5-1-2 is slightly smaller than the full-length 1.2 kb cHS4 element, and had no negative effect on vector titers in either orientation. This candidate contains a single CTCF/DHS site at the distal 3' end (Figure 5A), suggesting a much smaller functional element could be readily derived. Candidate 22-3 is already less than 0.3 kb, and also had no statistically significant effect on vector titer in one orientation, and only reduced vector titers 2.4-fold in the other orientation. Both elements were at least as effective as the full-length cHS4 element at blocking enhancer-promoter interactions in plasmid-based assay systems and at reducing the functional rate of gammaretroviral vector-mediated genotoxicity *in vivo*. As such, these elements provide two new options to the otherwise very limited arsenal of chromatin insulators validated for this purpose. Our studies also identified 10 other elements that appear to exhibit at least some enhancer-blocking insulator function, although we did not validate these elements in independent assays or assess them for transcriptional silencing activity. Nevertheless, none of these candidates, even the top two candidates, 5-1-2 or 22-3, exhibited enhancer-blocking insulator activity in excess of that seen with the prototypical cHS4 element. This supports the need for continued efforts to identify and characterize insulators from the human genome, both at the level of genomics and functional assays. Further, because we focused on candidates that are bound by CTCF, these studies are not capable of identifying other potential types of insulators.

We used a conventional gammaretroviral vector platform to assess the effects of new insulator candidates on vector-mediated genotoxicity and vector expression. Gammaretroviral vectors are the most common class of retroviral vectors used in clinical trials to date, are the most prone to inducing vector-mediated malignant transformation, and are the most sensitive to silencing position effects [1,33]. As such, gammaretroviral vectors provide the most sensitive tool for comparing the relative efficacy of different insulator elements, and would benefit the most from an expanded arsenal of validated chromatin insulators. Although the clinical gene therapy research community is increasingly utilizing lentiviral vectors with self-inactivating (SIN) long terminal repeats [33], there is evidence that chromatin insulators can reduce the rate of vector-mediated genotoxicity and the rate of silencing chromosomal position effects for this class of vectors as well [1]. There is also evidence that chromatin insulators can have negative effects on the titer of lentiviral-based vectors, with recent studies suggesting that these effects could be related to the size of the inserted fragment [34], or to sequences unique to the full-length cHS4 element [35]. Although lentiviral vectors are generally less sensitive to cryptic RNA processing signals and recombination between small stretches of homology, anecdotal reports of sequence incompatibilities also remain. As such, it is important to determine, on a case-by-case basis, the degree to which chromatin insulators or any other sequences affect the performance of individual vectors. By developing these two new validated insulators, we have expanded the available options for such vector optimization.

In order to efficiently test these new insulators, we first needed to develop a quantitative, robust, and efficient functional screen. The lentiviral vector-based GFP assay described here has many advantages in this regard. Issues of underlying gene transfer rates are resolved through highly efficient vector transduction and a cell-autonomous fluorescent reporter that can be analyzed by flow cytometry. The cloning is streamlined through the use of a single insertion site accessed by a rare-cutting restriction enzyme. It was uncertain whether this approach would work, since chromatin insulators are generally thought to work in pairs to form an insulated chromatin domain [2,11]. However, validation studies presented in Figure 4A demonstrate that the insertion of a single insulator element is sufficient in this setting to block enhancer-promoter interactions. The use of a fluorescent reporter also results in a more directly quantitative measure of reporter gene expression, and by extension, insulator activity, although this comes at the price of reduced sensitivity compared to the drug-resistant colony assay. Finally, this assay system requires less time than the drug-resistant colony assay, and is amenable to a higher degree of throughput, as evidenced by our parallel screening of 12 candidates. However, this assay also has a smaller dynamic range than the drug-resistant colony assay, and would be ineffective with candidate elements that are not compatible with the lentiviral vector life cycle.

The studies presented here, although designed to identify chromatin insulators for use in gene transfer vectors, also provide insights into the biology of chromatin insulators. First, the studies presented in Figure 4A indicate that the extended 400 bp core version of the cHS4 insulator, like the minimal 250 bp core, is less effective than the full-length 1.2 kb cHS4 fragment at blocking enhancer-promoter interactions. This finding is consistent with a recent report indicating that the 3' end of the 1.2 kb cHS4 fragment may also associate with CTCF and, when combined with the 5' 250 bp cHS4 core, reduces the rate of vector-mediated genotoxicity as effectively as the full-length fragment when assessed in culture [36]. Second, our studies serve to validate the general approach, reported by others [13], of using co-localization of DHSs and CTCF binding sites as a means of identifying potential enhancer-blocking insulators. Third, it appears that inclusion of candidates 5-1-2 and 22-3 in a gammaretroviral reporter vector marginally reduced the rate and level of vector silencing. This reduction in vector silencing is a hallmark of barrier insulator activity [1,10]. However, several lines of evidence indicate that the barrier activity of the prototypical cHS4 insulator is independent of CTCF binding [1,10]. Future studies will be needed to determine whether this anti-silencing activity is a general attribute of a subclass of CTCF-binding insulators or due to other sequences contained within the fragments used in these studies. Finally, our data point to an influence of orientation on the insulator activity of several candidate insulator elements, including the optimal candidates 5-1-2 and 22-3. Such orientation-dependency has also been reported for the cHS4 insulator here and in previous studies [32], suggesting that this may be a general and previously unrecognized property of chromatin insulators. Additional studies will be needed to determine whether these are common properties of chromatin insulators, as well as to identify the mechanisms underlying these phenomena.

## References

[1] Emery DW (2011) The use of chromatin insulators to improve the expression and safety of integrating gene transfer vectors. Hum Gene Ther 22: 761-774. doi:10.1089/hum.2010.233. PubMed: 21247248. 21247248 PMC3107579

[2] West AG, Gaszner M, Felsenfeld G (2002) Insulators: many functions, many mechanisms. Genes Dev 16: 271-288. doi:10.1101/gad.954702. PubMed: 11825869. 11825869

[3] Chung JH, Whiteley M, Felsenfeld G (1993) A 5' element of the chicken beta-globin domain serves as an insulator in human erythroid cells and protects against position effects in *Drosophila* . Cell 74: 505-514. doi:10.1016/0092-8674(93)80052-G. PubMed: 8348617. 8348617

[4] Emery DW, Yannaki E, Tubb J, Stamatoyannopoulos G (2000) A chromatin insulator protects retrovirus vectors from position effects. Proc Natl Acad Sci U S A 97: 9150-9155. doi:10.1073/pnas.160159597. PubMed: 10908661. 10908661 PMC16837

[5] Aker M, Tubb J, Groth AC, Bukovsky AA, Bell AC et al. (2007) Core sequences from the cHS4 insulator are necessary for protecting retroviral vectors from silencing position effects. Hum Gene Ther 18: 333-343. doi:10.1089/hum.2007.021. PubMed: 17411365. 17411365

[6] Ryu BY, Evans-Galea MV, Gray JT, Bodine DM, Persons DA et al. (2008) An experimental system for the evaluation of retroviral vector design to diminish the risk for proto-oncogene activation. Blood 111: 1866-1875. doi:10.1182/blood-2007-04-085506. PubMed: 17991809. 17991809 PMC2234041

[7] Arumugam PI, Higashimoto T, Urbinati F, Modlich U, Nestheide S et al. (2009) Genotoxic potential of lineage-specific lentivirus vectors carrying the beta-globin locus control region. Mol Ther 17: 1929-1937. doi:10.1038/mt.2009.183. PubMed: 19707188. 19707188 PMC2835044

[8] Li CL, Xiong D, Stamatoyannopoulos G, Emery DW (2009) Genomic and functional assays demonstrate reduced gammaretroviral vector genotoxicity associated with use of the cHS4 chromatin insulator. Mol Ther 17: 716-724. doi:10.1038/mt.2009.7. PubMed: 19240697. 19240697 PMC2835102

[9] Bell AC, West AG, Felsenfeld G (1999) The protein CTCF is required for the enhancer blocking activity of vertebrate insulators. Cell 98: 387-396. doi:10.1016/S0092-8674(00)81967-4. PubMed: 10458613. 10458613

[10] Gaszner M, Felsenfeld G (2006) Insulators: exploiting transcriptional and epigenetic mechanisms. Nat Rev Genet 7: 703-713. doi:10.1038/nrm2038. PubMed: 16909129. 16909129

[11] Phillips JE, Corces VG (2009) CTCF: Master weaver of the genome. Cell 137: 1194-1211. doi:10.1016/j.cell.2009.06.001. PubMed: 19563753. 19563753 PMC3040116

[12] Chung JH, Bell AC, Felsenfeld G (1997) Characterization of the chicken beta-globin insulator. Proc Natl Acad Sci U S A 94: 575-580. doi:10.1073/pnas.94.2.575. PubMed: 9012826. 9012826 PMC19555

[13] Xi H, Shulha HP, Lin JM, Vales TR, Fu Y et al. (2007) Identification and characterization of cell type-specific and ubiquitous chromatin regulatory structures in the human genome. PLOS Genet 3: e136. doi:10.1371/journal.pgen.0030136. PubMed: 17708682. 17708682 PMC1950163

[14] Yusufzai TM, Felsenfeld G (2004) The 5'-HS4 chicken beta-globin insulator is a CTCF-dependent nuclear matrix-associated element. Proc Natl Acad Sci U S A 101: 8620-8624. doi:10.1073/pnas.0402938101. PubMed: 15169959. 15169959 PMC423244

[15] Zychlinski D, Schambach A, Modlich U, Maetzig T, Meyer J et al. (2008) Physiological promoters reduce the genotoxic risk of integrating gene vectors. Mol Ther 16: 718-725. doi:10.1038/mt.2008.5. PubMed: 18334985. 18334985

[16] Ramezani A, Hawley TS, Hawley RG (2008) Combinatorial incorporation of enhancer-blocking components of the chicken beta-globin 5'HS4 and human T-cell receptor alpha/delta BEAD-1 insulators in self-inactivating retroviral vectors reduces their genotoxic potential. Stem Cells 26: 3257-3266. doi:10.1634/stemcells.2008-0258. PubMed: 18787211. 18787211 PMC2605779

[17] Gaussin A, Modlich U, Bauche C, Niederländer NJ, Schambach A et al. (2012) CTF/NF1 transcription factors act as potent genetic insulators for integrating gene transfer vectors. Gene Ther 19: 15-24. doi:10.1038/gt.2011.70. PubMed: 21562592. 21562592

[18] Pathak VK, Temin HM (1990) Broad spectrum of *in* *vivo* forward mutations, hypermutations, and mutational hotspots in a retroviral shuttle vector after a single replication cycle: deletions and deletions with insertions. Proc Natl Acad Sci U S A 87: 6024-6028. doi:10.1073/pnas.87.16.6024. PubMed: 2166940. 2166940 PMC54464

[19] Cavazzana-Calvo M, Payen E, Negre O, Wang G, Hehir K et al. (2010) Transfusion independence and HMGA2 activation after gene therapy of human beta-thalassaemia. Nature 467: 318-322. doi:10.1038/nature09328. PubMed: 20844535. 20844535 PMC3355472

[20] Tubb J, Groth AC, Leong L, Emery DW (2005) Simultaneous sequence transfer into two independent locations of a reporter vector using MultiSite Gateway technology. BioTechniques 39: 553-557. doi:10.2144/000112030. PubMed: 16235567. 16235567

[21] Lozzio CB, Lozzio BB (1975) Human chronic myelogenous leukemia cell line with positive Philadelphia chromosome. Blood 45: 321-324. PubMed: 163658. 163658

[22] Horn PA, Keyser KA, Peterson LJ, Neff T, Thomasson BM et al. (2004) Efficient lentiviral gene transfer to canine repopulating cells using an overnight transduction protocol. Blood 103: 3710-3716. doi:10.1182/blood-2003-07-2414. PubMed: 14739227. 14739227

[23] Zhang JW, Raich N, Enver T, Anagnou NP, Stamatoyannopoulos G (1990) Butyrate induces expression of transfected human fetal and endogenous mouse embryonic globin genes in GM 979 erythroleukemia cells. Dev Genet 11: 168-174. doi:10.1002/dvg.1020110207. PubMed: 2379328. 2379328

[24] Hawley RG, Lieu FH, Fong AZ, Hawley TS (1994) Versatile retroviral vectors for potential use in gene therapy. Gene Ther 1: 136-138. PubMed: 7584069. 7584069

[25] Aker M, Bomsztyk K, Emery DW (2010) Poly (ADP-ribose) polymerase-1 (PARP-1) contributes to the barrier activity function of a vertebrate chromatin insulator. J Biol Chem 285: 37589-37597. doi:10.1074/jbc.M110.174532. PubMed: 20876582. 20876582 PMC2988364

[26] Greenberger JS, Sakakeeny MA, Humphries RK, Eaves CJ, Eckner RJ (1983) Demonstration of permanent factor-dependent multipotent (erythoid/neutrophil/basophil) hematopoietic progenitor cell lines. Proc Natl Acad Sci U S A 80: 2931-2935. doi:10.1073/pnas.80.10.2931. PubMed: 6574462. 6574462 PMC393947

[27] Wang H, Maurano MT, Qu H, Varley KE, Gertz J et al. (2012) Widespread plasticity in CTCF occupancy linked to DNA methylation. Genome Res 22: 1680-1688. doi:10.1101/gr.136101.111. PubMed: 22955980. 22955980 PMC3431485

[28] Thurman RE, Rynes E, Humbert R, Vierstra J, Maurano MT et al. (2012) The accessible chromatin landscape of the human genome. Nature 489: 75-82. doi:10.1038/nature11232. PubMed: 22955617. 22955617 PMC3721348

[29] Bowers SR, Mirabella F, Calero-Nieto FJ, Valeaux S, Hadjur S et al. (2009) A conserved insulator that recruits CTCF and cohesin exists between the closely related but divergently regulated interleukin-3 and granulocyte-macrophage colony-stimulating factor genes. Mol Cell Biol 29: 1682-1693. doi:10.1128/MCB.01411-08. PubMed: 19158269. 19158269 PMC2655614

[30] Yannaki E, Emery DW, Stamatoyannopoulos G (2010) Gene therapy for beta-thalassaemia: the continuing challenge. Expert Rev Mol Med 12: e31. doi:10.1017/S1462399410001626. PubMed: 20883576. 20883576

[31] Hantzopoulos PA, Sullenger BA, Ungers G, Gilboa E (1989) Improved gene expression upon transfer of the adenosine deaminase minigene outside the transcriptional unit of a retroviral vector. Proc Natl Acad Sci U S A 86: 3519-3523. doi:10.1073/pnas.86.10.3519. PubMed: 2542934. 2542934 PMC287169

[32] Yannaki E, Tubb J, Aker M, Stamatoyannopoulos G, Emery DW (2002) Topological constraints governing the use of the chicken HS4 chromatin insulator in oncoretrovirus vectors. Mol Ther 5: 589-598. doi:10.1006/mthe.2002.0582. PubMed: 11991750. 11991750

[33] Rivière I, Dunbar CE, Sadelain M (2012) Hematopoietic stem cell engineering at a crossroads. Blood 119: 1107-1116. doi:10.1182/blood-2011-09-349993. PubMed: 22096239. 22096239 PMC3277348

[34] Bos TJ, de Bruyne E, van Lint S, Heirman C, Vanderkerken K (2010) Large double copy vectors are functional but show size-dependent decline in transduction efficiency. J Biotechnol 150: 37-40. doi:10.1016/j.jbiotec.2010.07.010. PubMed: 20638430. 20638430

[35] Urbinati F, Arumugam P, Higashimoto T, Perumbeti A, Mitts K et al. (2009) Mechanism of reduction in titers from lentivirus vectors carrying large inserts in the 3'LTR. Mol Ther 17: 1527-1536. doi:10.1038/mt.2009.89. PubMed: 19384292. 19384292 PMC2835256

[36] Arumugam PI, Urbinati F, Velu CS, Higashimoto T, Grimes HL et al. (2009) The 3' region of the chicken hypersensitive site-4 insulator has properties similar to its core and is required for full insulator activity. PLOS ONE 4: e6995. doi:10.1371/journal.pone.0006995. PubMed: 19746166. 19746166 PMC2736623

